# 한국 응급실 간호사의 계획된 행위이론 기반 환자안전간호활동 영향요인: 횡단적 조사연구

**DOI:** 10.7586/jkbns.26.025

**Published:** 2026-08-25

**Authors:** Sojung Park, Jihyun Kim

**Affiliations:** 1 Chungnam National University Hospital, Daejeon, Korea; 1 충남대학교 병원; 2 Department of Nursing, Daejeon University, Daejeon, Korea; 2 대전대학교 간호학과

**Keywords:** Patient safety, Emergency nursing, Nurses, Intention, Theory of planned behavior, 환자안전, 응급간호, 간호사, 의도, 계획된 행위이론

## Abstract

**Purpose:**

This study examined associations between the constructs of the Theory of Planned Behavior (TPB) and patient safety nursing activities among emergency department nurses. It also identified factors associated with these activities to provide foundational data for developing strategies to strengthen patient safety nursing practice.

**Methods:**

Participants were nurses working in emergency medical centers at seven general hospitals with more than 350 beds in the Daejeon–Chungcheong region. Data were collected from August 12 to August 30, 2024, and analyzed using descriptive statistics, analysis of variance, t-tests, Pearson correlation coefficients, and multiple regression.

**Results:**

The mean score for patient safety nursing activities was 4.43 out of 5. The mean TPB construct scores were 6.39 out of 7 for attitude, 6.48 for subjective norm, 5.10 for perceived behavioral control, and 5.65 for behavioral intention. Patient safety nursing activities were positively correlated with attitude (r = .55, *p* < .001), subjective norm (r = .55, *p* < .001), perceived behavioral control (r = .59, *p* < .001), and behavioral intention (r = .65, *p* < .001). Multiple regression identified behavioral intention (β = .37, *p* < .001), subjective norm (β = .21, *p* = .002), attitude (β = .16, *p* = .017), perceived behavioral control (β = .15, *p* = .048), and age of 30 to < 35 years (β = .18, *p* = .010) as significant explanatory factors. The model explained 63% of the variance in patient safety nursing activities (F = 22.21, *p* < .001).

**Conclusion:**

The findings support the applicability of the TPB for explaining patient safety nursing activities among emergency department nurses. Educational interventions and organizational strategies that strengthen behavioral intention, subjective norm, attitude, and perceived behavioral control may help improve patient safety nursing activities and support safer clinical practice.

## 서론

### 1. 연구의 필요성

환자안전은 의료서비스 제공의 기본이며 의료의 질을 결정하는 데 있어 중요한 측면을 구성하고 있다[1]. 환자안전을 위한 국제분류(International Classification for Patient Safety)에서 환자안전은 ‘의료-병원 과정 전반에 걸쳐 발생하는 불리한 결과나 부상을 방지, 예방 또는 개선하는 행위’로 정의하고 있다[2]. 환자안전사고는 환자의 생명에 위협과 재원기간의 연장은 물론 의료진에 대한 신뢰감 저하와 의료의 질 저하[3] 뿐만 아니라 국가의 재정적 손실 등을 발생시킨다[2].

응급실에서의 환자안전사고 보고 건수는 2020년 260건에서 2023년 367건으로 꾸준히 증가하는 추세이다[4]. 응급실은 생명이 위급한 환자에게 짧은 시간 동안 집중적인 의료서비스를 제공하는 곳으로, 불확실하고 예측 불가능한 상황에서 갑작스러운 사건이 자주 발생할 수 있어 병원 내 부서 중 안전사고 발생 비율이 가장 높은 곳이다[5,6]. 또한 응급실 내원 환자 수는 지속적으로 증가하는 추세로 2022년 기준 8,874,564명으로 2년 사이 약 12%가 증가하였다[4]. 이 같은 응급실 방문 환자 수의 증가와 입원 병상 부족으로 인한 응급실에서의 체류시간 연장은 응급실의 과밀화를 심화시켜 간호사의 환자에 대한 직접 간호 시간의 감소와 환자안전사고 발생을 높이는 요인이 된다[7]. 간호사는 환자안전활동과 관련하여 큰 비중을 담당하고 모니터링과 감시, 직접적 환자안전간호활동을 통해 환자안전을 보장하는 중요한 역할을 수행한다[8]. 이에 응급실에서의 환자안전사고 발생 감소를 위해서는 응급실 간호사들의 환자안전간호활동에 관한 연구와 증진 전략 수립이 필요하다.

계획된 행위이론은 개인의 행위를 예측하고 설명할 수 있는 개념적 모델로서[9], 응급실 간호사의 환자안전간호활동 이행을 체계적으로 예측하고 이를 기반으로 환자안전간호활동의 증진 방안을 제시할 수 있다. 계획된 행위이론에서는 행위에 대한 태도, 주관적 규범, 지각된 행위통제, 행위의도를 주요 영향요인으로 설명하였으며, 행위에 대한 태도는 특정 행위를 수행한다는 것이 긍정적 또는 부정적으로 어떤 가치가 있는지를 의미한다[9]. 주관적 규범이란 특정 행위 수행에 대해 개인이 주관적으로 지각하는 사회적 압력을 의미하며, 지각된 행위통제는 어떤 행위를 실제로 수행할 능력에 대한 인식을 의미한다[9]. 의도는 행위에 대한 선행요인으로, 특정 행위를 실행하기 위한 개인의 준비 상태를 반영하는 지표이다[9]. 계획된 행위이론에서 제시된 주요 영향요인은 손위생 행위[10], 의료관련 감염관리지침 수행[11,12], 항암제 관리지침 이행[13] 등 다양한 보건의료 분야 연구에서 행위를 예측하는 변수로 적용되었다. 응급실은 긴박한 상황 속에서 짧은 시간 내 빠른 판단과 행위가 요구되는 환경적 특성을 지닌다. 이러한 특성으로 인해 간호사의 태도, 지각된 행위통제, 주관적 규범, 행위의도가 실제 환자안전간호활동에 미치는 영향이 클 수 있다고 예측된다. 계획된 행위이론은 이처럼 즉각적인 의사결정과 행위가 요구되는 환경에서 개인의 내·외적 요인이 행위에 미치는 영향을 체계적으로 설명하는 데 적합한 이론으로, 응급실 간호사의 환자안전간호활동을 이해하고 그 영향요인 간의 관계를 파악하며 행위를 예측하는 데 효과적으로 적용될 수 있다. 또한 국내에서 계획된 행위이론을 기반으로 응급실 간호사의 환자안전간호활동을 분석한 연구는 매우 제한적이므로, 본 연구에서 이 이론을 적용하는 것은 이론적·실무적으로 의의가 있다.

환자안전간호활동에 대한 선행연구는 안전관리 현황 또는 지식, 인식, 환자안전문화 등의 영향요인을 단편적으로 파악하거나 이행과의 관계를 분석한 연구[14-18]가 대부분으로 환자안전간호활동의 선행요인을 총체적으로 이해하고 이행 행위에 미치는 영향을 파악하는 데는 한계가 있다.

이에 본 연구는 계획된 행위이론을 바탕으로 응급실 간호사의 환자안전간호활동에 영향을 미치는 복합적 선행 요인을 파악하여 응급실 간호사의 환자안전간호활동 향상을 위한 중재 전략 개발의 자료를 제공하고자 한다.

### 2. 연구의 목적

본 연구는 계획된 행위이론을 기반으로 응급실 간호사의 환자안전간호활동과 계획된 행위이론 구성요인 간의 관련성을 파악하고, 환자안전간호활동의 설명 요인을 확인하고자 하였다. 이를 위해 첫째, 대상자의 일반적 특성 및 직무 관련 특성에 따른 환자안전간호활동의 차이를 파악하고, 둘째, 태도, 주관적 규범, 지각된 행위통제, 행위의도 및 환자안전간호활동의 수준을 확인하며, 셋째, 이들 변수 간 상관관계를 분석하고, 넷째, 환자안전간호활동의 설명 요인을 확인하였다.

## 연구 방법

### 1. 연구 설계

본 연구는 계획된 행위이론을 기반으로 응급실 간호사의 환자안전간호활동에 영향을 미치는 요인을 파악하기 위한 서술적 횡단적 조사연구이다. 본 연구는 관찰연구 보고지침인 STROBE (Strengthening the Reporting of Observational Studies in Epidemiology) statement에 근거하여 연구 내용을 보고하였다.

### 2. 연구 대상

본 연구의 대상자는 대전·충청권 소재 350병상 이상 규모의 종합병원 7개 응급의료센터(충남대병원, 건양대병원, 대전성모병원, 을지대학교병원, 한국병원, 유성선병원, 대전보훈병원)에 근무하는 간호사이다. 해당 지역은 권역·지역응급의료센터가 고르게 분포하여 응급실 간호 인력의 근무 환경을 대표성 있게 반영할 수 있어 연구 대상으로 선정하였다. 구체적으로 임상경력 3개월 이상의 간호사 중 응급실에서 독립적인 간호를 수행하는 간호사를 대상으로 하였으며, 직접적으로 환자간호를 수행하지 않는 간호관리자는 제외하였다.

적정 표본 수는 선행연구[19]를 근거로 G*Power 3.1.9.7 프로그램을 이용하여 산출하였다. 유의수준 .05, 중간 효과크기 .15, 검정력 .80, 예측변수 15개를 기준으로 계산한 결과 최소 139명이 필요하였다. 국내 간호사 대상 선행연구의 탈락률을 근거로[20], 본 연구의 탈락률은 15%로 선정하였으며 최소 164부가 필요하였으나 본 연구에서는 이를 상회하는 167부를 배부·회수하였다. 최종 응답이 불성실한 15부를 제외한 152부를 분석에 사용하였다.

### 3. 연구 도구

#### 1) 일반적 특성 및 직무관련 특성

일반적 특성과 직무관련 특성은 국내 간호사의 환자안전간호활동 관련 선행연구[21-23]를 근거로 선정하였으며, 성별, 연령, 교육, 결혼 유무, 직무관련 특성으로 근무하는 응급실 종별, 총 임상 경력, 응급실 경력, 최근 1년 이내 안전교육 유무와 횟수, 사건보고 체계 인지 여부, 사건 보고 경험 여부를 포함한 11문항으로 이루어져 있다.

#### 2) 환자안전간호활동

환자안전간호활동 측정도구는 2014년도 의료기관평가인증원에서 개발한 병원인증평가 항목을 바탕으로 Han과 Jung [24]이 수정·보완한 도구를 사용하였다. 도구는 정확한 환자확인, 수술/시술 전 환자안전, 의사소통, 낙상예방활동, 손위생 및 감염관리, 투약, 화재안전 및 응급상황관리, 시설 및 의료기기 관리의 총 32문항으로 구성되었다. Likert 5점 척도로, 점수가 높을수록 환자안전간호활동 정도가 높음을 의미한다. 도구의 신뢰도는 Han과 Jung [24]의 연구에서 Cronbach’s α .95이었으며, 본 연구에서는 .94이었다.

#### 3) 환자안전간호활동에 대한 태도

환자안전간호활동에 대한 태도를 측정하기 위해 Ajzen [9]의 ‘Constructing a Theory of Planned Behavior Questionnaire’와 Moon과 Song [11]의 도구를 기반으로 Kim [3]이 수정·보완한 도구를 사용하였다. 도구는 총 4개 문항으로 7점 척도의 어의 구별 척도이다. 점수가 높을수록 환자안전간호활동에 대한 태도가 긍정적임을 의미한다. 도구의 신뢰도는 Kim [3]의 연구에서 Cronbach’s α .77이었고, 본 연구에서는 .71이었다.

#### 4) 환자안전간호활동에 대한 주관적 규범

환자안전간호활동에 대한 주관적 규범을 측정하기 위해 Ajzen [9]의 ‘Constructing a Theory of Planned Behavior Questionnaire’와 Moon과 Song [11]의 도구를 기반으로 Kim [3]이 수정·보완한 도구를 사용하였다. 본 도구는 총 5문항으로 Likert 7점 척도이며 점수가 높을수록 환자안전간호활동을 수행하도록 하는 사회적 압력을 크게 느끼는 것을 의미한다. 도구의 신뢰도는 Kim [3]의 연구에서 Cronbach’s α .91이었고, 본 연구에서는 .85이었다.

#### 5) 환자안전간호활동에 대한 지각된 행위통제

환자안전간호활동에 대한 지각된 행위통제를 측정하기 위해 Ajzen [9]의 ‘Constructing a Theory of Planned Behavior Questionnaire’와 Moon과 Song [11]의 도구를 기반으로 Kim [3]이 수정·보완한 도구를 사용하였다. 본 도구는 총 5문항으로 Likert 7점 척도로 측정한다. 점수가 높을수록 지각된 행위통제가 높음을 의미한다. 도구의 신뢰도는 Kim [3]의 연구에서 Cronbach’s α .88이었고, 본 연구에서는 .89이었다.

#### 6) 환자안전간호활동에 대한 행위의도

환자안전간호활동에 대한 행위의도를 측정하기 위해 Ajzen [9]의 ‘Constructing a Theory of Planned Behavior Questionnaire’와 Moon과 Song [11]의 도구를 기반으로 하여 Kim [3]이 수정·보완한 도구를 사용하였다. 이 도구는 환자안전간호활동 수행에 대한 대상자의 의도나 계획을 측정하는 것으로 총 5문항으로 Likert 7점 척도로 측정한다. 점수가 높을수록 환자안전간호활동에 대한 행위의도가 높음을 의미한다. 도구의 신뢰도는 Kim [3]의 연구에서 Cronbach’s α .90이었고, 본 연구에서는 .89이었다.

### 4. 자료 수집 및 분석

본 연구의 자료 수집은 2024년 08월 12일부터 2024년 08월 30일까지 진행하였으며, 대전·충청권에 소재한 350병상 이상의 7개 종합병원 간호부를 방문하여 연구목적과 자료조사 방법을 설명하고 승인을 받은 뒤 연구를 진행하였다. 설문지의 배포와 수거는 연구자가 직접 시행하였으며 설문 소요 시간은 15~20분 이내였다.

수집된 자료는 SPSS version 28.0 (IBM Corp., Armonk, NY, USA) 프로그램을 이용하여 분석하였다. 대상자의 특성과 변수의 수준은 빈도, 백분율, 평균, 표준편차로 분석하였다. 대상자의 특성에 따른 변수의 차이는 independent t-test와 one-way analysis of variance, Scheffé’s test로 분석하였으며, 변수 간 상관관계는 Pearson correlation으로 분석하였다. 환자안전간호활동에 영향을 미치는 요인은 다중회귀분석으로 분석하였다.

### 5. 윤리적 고려

본 연구는 대전대학교의 생명의학연구윤리심의위원회의 심의 승인(IRB 1040647-202406-HR-026-01)을 받은 후 각 종합병원 간호부의 승인을 받고 연구를 진행하였다. 연구대상자에게 설문 시작 전 연구의 목적과 방법, 자발적 참여 동의, 자료의 비밀보장, 연구철회 가능성, 연구 참여에 따른 불이익이 없음에 대해 설명하고 서면 동의를 받았다. 연구에 필요한 정보만을 수집하였으며, 본 연구의 참여로 수집된 연구대상자의 신상정보는 비밀 상태로 유지되었고 수집된 정보는 연구 목적 외에는 사용되지 않았다. 연구 종료 후 설문자료는 3년 보관하고 폐기될 예정이다.

## 연구 결과

### 1. 대상자의 특성 및 대상자 특성에 따른 환자안전간호활동의 차이

본 연구의 대상자는 여자가 121명(79.6%), 남자가 31명(20.4%)이었다. 학력은 4년제 졸업이 127명(83.6%)으로 가장 많았으며 결혼 여부는 미혼 116명(76.3%), 기혼 36명(23.7%)이었다. 총 병원 근무 경력은 평균 6.4 ± 4.7년으로, 5년 미만이 69명(45.4%)으로 가장 많았다. 응급실 근무 경력은 평균 5.4 ± 3.6년이었으며, 5년 미만이 83명(54.6%)으로 가장 많았다. 1년 이내 안전관리교육 경험은 ‘없음’이 19명(12.5%), ‘있음’이 133명(87.5%)이었다(Table 1).

대상자의 특성에 따른 환자안전간호활동의 차이를 분석한 결과, 연령(F = 10.44, *p* < .001), 결혼 유무(t = -2.21, *p* = .028), 병원 근무 경력(F = 8.86, *p* < .001), 응급실 근무 경력(F = 5.66, *p* = .004), 1년 이내 안전관리교육 경험(t = -2.39, *p* = .018), 교육횟수(F = 3.16, *p* = .026), 1년 이내 본인이 일으킨 환자안전사건보고 경험(t = 2.27, *p* = .025)에 따라 환자안전간호활동은 통계적으로 유의미한 차이를 보였다(Table 1).

### 2. 대상자의 환자안전간호활동에 대한 태도, 주관적 규범, 지각된 행위통제, 행위의도 및 환자안전간호활동

대상자의 환자안전간호활동에 대한 태도, 주관적 규범, 지각된 행위통제, 행위의도 및 환자안전간호활동을 분석한 결과는 다음과 같다(Table 2).

대상자의 환자안전간호활동에 대한 태도에 대한 평균점수는 7점 만점에 6.39 ± 0.70점이었으며, 주관적 규범의 평균점수는 7점 만점에 6.48 ± 0.60점이었고, 지각된 행위통제의 평균점수는 7점 만점에 5.10 ± 1.10점, 행위의도는 7점 만점에 5.65 ± 0.98점, 환자안전간호활동에 대한 평균점수는 5점 만점에 4.43 ± 0.44점으로 나타났다.

환자안전간호활동의 하위영역을 분석한 결과, 투약의 평균점수는 5점 만점에 4.57 ± 0.44점, 정확한 환자확인 4.56 ± 0.54점, 손위생 및 감염관리 4.55 ± 0.49점, 화재안전 및 응급상황관리 4.37 ± 0.60점, 시설 및 의료기기관리 4.36 ± 0.73점, 낙상예방활동 4.30 ± 0.59점, 수술·시술 전 환자확인 4.30 ± 0.75점, 의사소통 4.28 ± 0.65점 순으로 나타났다.

### 3. 대상자의 환자안전간호활동과 환자안전간호활동에 대한 태도, 주관적 규범, 지각된 행위통제 및 행위의도의 상관관계

변수 간 상관관계를 분석한 결과 환자안전간호활동은 환자안전간호활동에 대한 태도(r = .55, *p* < .001), 환자안전간호활동에 대한 주관적 규범(r = .55, *p* < .001), 환자안전간호활동에 대한 지각된 행위통제(r = .59, *p* < .001), 환자안전간호활동에 대한 행위의도(r = .65, *p* < .001)와 모두 통계적으로 유의한 상관관계를 나타냈다(Table 3).

### 4. 대상자의 환자안전간호활동에 미치는 영향요인

대상자의 환자안전간호활동에 영향을 미치는 요인을 확인하기 위해 일반적 및 직무관련 특성 중 환자안전간호활동에 유의한 차이를 보였던 변수, 연령, 결혼 여부, 병원 근무경력, 응급실 근무경력, 안전관리교육 경험, 안전관리교육 횟수, 환자안전사건보고 경험을 통제변수로 투입하였다. 회귀분석의 기본 가정 충족 여부를 검토하기 위해 다중공선성 및 잔차의 독립성, 선형성, 잔차의 정규성 및 등분산성, 이상값 및 영향점을 확인하였다. 공차(tolerance)는 0.26∼0.93으로 0.1 이상이었고, 분산팽창계수(VIF)는 1.07∼3.85로 모두 10 미만으로 다중공선성의 문제가 없었다. 투입변수 중 병원 근무경력과 응급실 근무경력은 다른 변수들에 비해 분산팽창계수가 상대적으로 높게 나타났으나(각각 3.85, 2.53), 허용 기준(VIF < 10) 이내였다. 두 변수는 전체 임상경력과 응급실이라는 특정 부서에서의 경력이라는 점에서 개념적으로 구분되는 정보를 제공한다고 판단하여 모형에 함께 투입하였다. 총 병원 근무경력은 임상 전반에서 축적된 숙련도를, 응급실 근무경력은 중증도와 긴급성이 높은 응급실이라는 특수 부서에서의 경험을 반영하므로, 두 변수를 함께 투입함으로써 전반적 임상 숙련도와 부서 특수적 경험의 효과를 구분하여 통제하고자 하였다. Durbin-Watson 통계값은 1.88로 자기 상관에 문제가 없었다. 표준화 잔차와 표준화 예측값의 산점도를 검토한 결과 잔차가 특정한 패턴 없이 무작위로 고르게 분포하여 선형성과 등분산성의 가정을 충족하였다. 정규 P-P plot 검토 결과 잔차가 대각선에 근접하여 분포함으로써 정규성 가정을 만족하였다. Cook's distance는 0.00∼0.18의 범위로 모두 기준값 1.0 미만이었고 leverage 값 검토에서도 회귀계수 추정에 과도한 영향을 미치는 사례는 없었으며, 표준화 잔차의 절댓값이 3을 초과하는 사례는 없어 이상값 및 영향점의 문제는 없는 것으로 확인되었다. 다중회귀분석 결과, 연령(30세 이상 35세 미만, β = .18, *p* = .010), 환자안전간호활동에 대한 행위의도(β = .37, *p* < .001), 주관적 규범(β = .21, *p* = .002), 태도(β = .16, *p* = .017), 지각된 행위통제(β = .15, *p* = .048)가 유의한 설명변수로 분석되었으며, 회귀모형의 수정된 설명력은 63%로 나타났다(F = 22.21, *p* < .001)(Table 4).

## 논의

본 연구는 계획된 행위이론을 기반으로 응급실 간호사의 환자안전간호활동 정도를 파악하고 주요 설명 요인을 확인하고자 시행되었다.

본 연구에서 응급실 간호사의 환자안전간호활동은 5점 만점에 4.43점이었다. 이 같은 결과는 종합병원 간호사를 대상으로 같은 도구를 사용하여 측정한 Han과 Jung [24]의 연구에서 환자안전간호활동이 4.05점이었던 결과보다 다소 높은 수준을 나타냈다. 이는 2011년 환자안전과 의료의 질 향상을 위하여 시작된 의료기관 인증평가의 지속적 시행과 관련된 것으로 분석된다. 각 의료기관은 의료기관 평가인증 기준에 따라 의료의 질 향상과 환자안전 활동의 수행을 위한 전담 부서를 필수로 지정하고 지속적 교육과 활동상태를 점검하고 있다[25]. 또한 환자안전 관련 규정 설정, 사고의 재발 방지를 위한 개선 권고사항 및 예방 활동 사례 등을 포함한 교육 자료를 공유하고 있다[25]. 이 같은 주기적인 의료기관 인증평가를 통해 환자안전간호활동에 대한 체계를 확립하였고 이를 통해 의료기관의 환자안전간호활동이 점차 증가한 것으로 분석된다. 그러나 환자안전간호활동의 하위영역별 수준을 분석한 결과, 투약이 4.57점으로 가장 높았으며 다음으로 정확한 환자확인 4.56점, 손위생 및 감염관리 4.55점, 화재안전 및 응급상황관리 4.37점, 시설 및 의료기기관리 4.36점, 낙상예방활동 4.30점, 수술·시술 전 환자확인 4.30점, 의사소통 4.28점 순으로 나타나 영역별 차이가 있음을 확인할 수 있다. 의사소통 영역은 환자안전간호활동 하위영역 중 가장 낮게 나타났으며 이는 환자안전을 위한 의사소통이 다른 안전간호활동에 비해 상대적으로 미흡하게 이루어지고 있음을 시사한다. 이러한 결과는 종합병원간호사 대상 선행연구[26]에서 의사소통 영역의 점수가 가장 낮게 나타난 결과와 유사하다. 의사소통의 문제는 환자안전을 위협하는 요소로, 특히 복잡한 상황에서 신속한 의사결정 능력이 요구되는 응급실 간호사의 경우 의사소통의 문제는 주요한 환자안전문제를 유발할 수 있다. 이에 간호환경의 특수성을 고려한 환자안전간호활동 관련 표준화된 의사소통 지침이 마련되어져야 한다. 선행연구에 따르면 표준화된 의사소통 체계의 도입은 간호사의 환자안전에 대한 인식과 태도를 긍정적으로 변화시키고, 환자안전간호활동의 실천을 촉진하는 것으로 확인되었다[27]. 응급실은 환자의 많은 입·퇴원과 빈번한 타과 협진 등으로 의사소통 오류 가능성이 높은 환경임을 고려할 때, 단순한 지침 개발을 넘어 SBAR (Situation-Background –Assessment-Recommendation, 상황-배경-사정-제안) 기반의 표준화된 환자 인계 체계[27] 도입이 필요하며 응급실과 타 부서 간 실시간 정보 공유가 가능한 다학제 간 의사소통 프로토콜을 마련하는 등 응급실 환경의 특수성을 반영한 구체적인 실무 적용 방안 마련이 필요하다.

본 연구에서 대상자의 특성에 따른 응급실 간호사의 환자안전간호활동은 연령, 총 병원근무 경력, 응급실 근무 경력, 안전관리 교육 경험, 안전관리 교육 횟수에 따라 유의한 차이를 나타냈다. 근무 경력이 높을수록 환자안전간호활동의 수행이 높은 결과는 중환자실 간호사의 안전간호활동을 분석한 선행연구 결과[28]와 유사한 결과이다. 그러나 다중회귀분석을 통한 영향요인 분석에서 총 병원근무 경력과 응급실 근무 경력 모두 유의한 영향요인으로 확인되지 않아, 경력이 환자안전간호활동에 미치는 영향에 대해서는 추후 후속 연구를 통한 검증이 필요하다. 또한 본 연구에서 최근 1년 이내 안전교육 경험이 있는 경우에서 수행 정도가 높은 결과는 신규간호사를 대상으로 감염관리 교육을 시행한 후 감염관리 수행 정도가 향상된 결과[29]와 교육 경험이 있는 경우 안전간호활동 수행도가 높았던 선행연구 결과[28]와 일맥상통한다. 이처럼 교육 중재는 환자안전간호활동을 증진시킬 수 있는 중재 방법으로 정기적으로 적용되어야 한다. 또한 환자안전간호활동의 영역 중 하나인 낙상 및 감염관리 교육 중재 연구에서 임상 상황 시나리오기반 시뮬레이션 교육이 지식과 태도, 관련 행위를 증진시키는 데 효율적인 것으로 보고되었다[23,29]. 이에 임상현장에서 간호사들이 효과적으로 활용할 수 있는 시나리오 개발과 적용을 위한 연구가 이루어져야 할 것이다.

응급실 간호사의 환자안전간호활동에 영향요인을 분석한 결과, 연령, 환자안전간호활동에 대한 태도, 주관적 규범, 지각된 행위통제, 행위의도가 유의한 영향요인으로 나타났다. 연령은 30세 미만 집단을 기준으로 30세 이상 35세 미만 집단에서만 통계적으로 유의하였으며(β = .18, *p* = .010), 35세 이상 집단은 유의하지 않았다(β = .15, *p* = .066). 이 같은 결과는 일반병동 간호사를 대상으로 전문성과 임상수행능력을 분석한 Rabie [30]의 연구와 차이를 보이는 결과이다. 선행 연구[30]에서도 연령은 간호사의 전문성과 임상수행능력의 주요 영향요인으로 나타났으나 연령이 증가할수록 전문성과 임상수행능력이 증가하는 것으로 나타났다. 그러나 본 연구에서는 35세 이상 집단에서 유의한 차이가 확인되지 않아 연령과 환자안전간호활동 간의 관계가 단순한 선형관계로 설명하는데 한계가 있다. 이 같은 연구결과를 바탕으로 응급실 간호사의 환자안전간호활동 향상을 위한 교육적 접근이 연령대에 따라 차별화될 필요가 있음을 알 수 있다. 획일적인 교육프로그램보다는 연령대별 특성을 반영한 환자안전간호활동 교육프로그램 개발이 필요하며, 연령대 자체가 갖는 고유한 특성(생애주기별 역할 부담, 위험지각 수준의 차이 등)에 초점을 맞춘 교육 설계가 필요하다. 또한 본 연구에서 35세 이상 집단의 표본 수가 상대적으로 적어 해당 연령군에서 통계적 검정력이 제한적이었을 가능성을 배제할 수 없다. 이에 연령대별 층화표집을 통해 각 연령군의 표본 수를 균형 있게 확보한 후 연령과 환자안전간호활동 간의 관계를 검증하는 후속 연구가 필요하다.

본 연구결과 응급실 간호사의 환자안전간호활동에 간호사들의 환자안전간호활동에 대한 태도가 영향을 미치는 것으로 분석되었다. 이는 대학병원 간호사를 대상으로 한 선행연구[23]와 중환자실 간호사를 대상으로 한 연구[28]의 결과와 유사한 것이다. 다수의 선행연구[23,28,31]에서 태도는 행위에 영향을 미치는 주요 요인으로 행위에 대한 긍정적 태도가 행위를 증진시키는 것으로 보고되었다. 이에 응급실 간호사들의 환자안전간호활동 증진을 위해서는 응급상황에서의 환자안전간호활동에 대한 부정적 인식을 개선하는 방향의 교육이 필요하다. 또한 본 연구에서 응급실 간호사들의 환자안전간호활동에 대한 태도는 7점 만점에 6.39점이었으며 이는 수술실 간호사를 대상으로 한 Kim [32]의 연구에서 6.00점으로 나타난 것과 유사한 결과이다. 행위에 대한 긍정적 태도는 행위를 증진시키는 요인으로 선행연구[23,33]에서 행위에 대한 태도는 행위에 대한 지식이 향상될수록 긍정적으로 변화된다고 보고하였다. 실제 간호대학생을 대상으로 환자안전 기반 시뮬레이션 실습교육 중재를 시행하고 효과를 검증한 선행연구[23]에서 환자안전 관련 지식의 향상은 환자안전 관련 태도와 환자안전관리 활동을 증진시키는 것으로 보고되었다. 이에 환자안전간호활동에 대한 체계적 교육을 통해 간호사들의 지식을 향상시키고 이를 바탕으로 환자안전간호활동 수행에 대한 태도를 긍정적으로 변화시키는 것이 필요하다.

본 연구에서 주관적 규범은 응급실 간호사의 환자안전간호활동의 주요한 영향요인으로 분석되었다. 이는 선행연구 중환자실 간호사를 대상으로 한 Yoon [28]의 연구결과와 유사한 결과이다. 또한 본 연구에서 환자안전간호활동에 대한 주관적 규범은 7점 만점에 6.48점으로 높은 점수를 나타냈으며, 이는 Kim [32]의 연구에서 수술실 간호사의 주관적 규범이 6.00점으로 나타난 결과와 유사한 수준이다. 주관적 규범은 특정한 행위의 수행에 대한 사회적 압력을 개인이 인식하는 정도를 의미하며, 주관적 규범이 높을수록 행위를 실천하려는 의도가 높아지고, 이는 실제 행동으로 이어질 가능성이 높다[9]. 본 연구와 선행연구의 결과를 통해 간호사들이 환자안전간호활동에 대한 사회적 압력을 높게 인식하고 있는 것을 알 수 있으며, 주관적 규범은 환자안전간호활동과 유의한 정적 상관을 보이는 것으로 나타났다. 이는 2016년 환자안전법이 시행되면서 환자안전간호활동에 대한 사회적 인식의 증가와 의료기관 인증평가 시행을 통한 환자안전간호활동의 중요성 강조에 따라[25] 사회적 압력이 증가되면서 주관적 규범이 향상된 것으로 분석된다[34,35]. 또한 주관적 규범의 향상은 환자안전간호활동과 같이 행동의 변화를 유도하는 중재 연구들에서 주요한 중재 전략 변인으로 다루어져야 할 것이다.

본 연구에서 응급실 간호사의 환자안전간호활동에 대한 지각된 행위통제는 환자안전간호활동의 주요 영향요인으로 분석되었다. 이는 중환자실 간호사를 대상으로 한 Gu 등[36]과 수술실 간호사를 대상으로 한 Lee [10]의 연구와 유사한 결과이다. 지각된 행위통제는 어떠한 행위에 대해 개인이 방해되는 요인을 스스로 통제할 수 있다고 인식하는 정도를 말하며[9], 특정 행위의 증진을 위해서는 지각된 행위통제의 향상이 선행되어야 한다. 응급실 간호사가 실무에서 환자안전간호활동을 이행하는 것에 대한 어려움을 느끼더라도 방해요인을 스스로 통제할 수 있다는 인식을 향상시키는 것이 강조되어야 하며 이를 위한 프로그램이나 교육의 적용이 필요하다. 또한 본 연구에서 환자안전간호활동에 대한 지각된 행위통제는 7점 만점에 5.10점으로 중상 정도 수준이었으며, 이는 수술실 간호사를 대상으로 한 Kim [32]의 4.95점과 유사한 점수이다. 실제적인 행위통제는 자원이나 기회의 이용가능성과 관련되며[9], 본 연구에서는 응급실의 환경적 요인의 개선과 관련될 수 있다. 실제 선행연구에서 간호사는 의료기관의 지침 이행의 방해요인을 스스로 통제하여 지침을 충분히 이행할 수 있다고 지각하지만 담당 환자 수[37]와 간호업무량[38] 등과 같이 개인이 통제할 수 없는 요인이 행위통제에 대한 지각에 영향을 미치는 것으로 분석되었다. Ajzen [9]은 지각된 행위통제를 행위의도에 선행하는 변수로 규정하였으며, 실제적인 행위통제가 정확히 반영될 때 행위에 직접적인 영향을 미칠 수 있다고 제시하였다. 이를 근거로 시설, 인력, 물품 지원 등 실무 환경적 요인이 개선되지 않는 한 지각된 행위통제의 향상은 기대하기 어려우며, 이에 기반한 환자안전간호활동의 증진 또한 제한될 수밖에 없다. 이에 응급실 간호사의 환자안전간호활동에 대한 지각된 행위통제를 향상시키기 위해서는 체계적인 교육 및 중재 프로그램의 적용과 함께, 시설 및 인력 지원을 포함한 조직 수준의 환경적 지원 체계 구축이 요구된다.

본 연구에서 응급실 간호사의 환자안전간호활동에 대한 행위의도는 간호사들의 환자안전간호활동의 주요 영향요인으로 분석되었으며 이는 행위의도가 행위의 주요 선행요인이라는 Ajzen [9]의 주장을 지지하는 결과이다. 또한 행위의도가 수술실 간호사의 환자안전간호활동의 예측요인으로 분석된 결과[32]와 일치하며, 병동 간호사를 대상으로 한 Roh와 Kim [13]의 연구와 간호대학생을 대상으로 한 Kim [3]의 연구에서 행위의도가 행위 수행에 영향을 미친다는 결과와 일맥상통한다. 행위의도는 선행연구[39]를 통해 특정 행위 이행과 가장 가깝게 관련되어 있는 기질로 보고되었으며, 본 연구에서도 환자안전간호활동의 가장 큰 영향 요인으로 나타났다. 또한 본 연구에서 환자안전간호활동에 대한 행위의도는 7점 만점에 5.65점으로 나타났으며 이는 수술실 간호사를 대상으로 한 Kim [32]의 5.55점과 유사한 점수이다. 본 연구와 선행연구 결과를 바탕으로 행위의도의 향상은 실질적인 행위를 증진시킬 수 있는 방안으로 이를 위한 체계적인 노력이 필요하다. 다수의 선행연구[3,13,37,40,41]에서 행위에 대한 지식의 향상은 행위에 대한 태도, 주관적 규범, 지각된 행위통제가 향상을 유발하고 이를 근거로 행위의도가 높아진다고 보고하였다. 따라서 응급실 간호사의 환자안전간호활동에 대한 교육을 통한 지식의 향상과 이를 기반으로 한 환자안전간호활동에 대한 행위의도의 향상이 필요하다. 또한 행위의도와 근무 경력과의 관련성을 분석한 연구에서 근무 경력이 많은 숙련된 간호사의 행위의도가 상대적으로 더 높은 것으로 보고하였다[42]. 이는 경력이 많은 간호사는 숙련된 전문가로서 경험적 지식과 직관을 통해 응급상황에 능숙하게 대처하여 효율적인 간호 결과를 환자에게 제공할 수 있으며, 이 같은 경력간호사의 전문성은 환자안전간호활동에 대한 행위의도를 형성하는 주요 요인으로 작용한다[43,44].

본 연구는 계획된 행위이론을 기반으로 응급실 간호사의 환자안전간호활동에 영향을 미치는 요인을 체계적으로 규명하였다는 점에서 의의를 갖는다. 환자안전간호활동을 구성하는 정확한 환자확인, 투약, 손위생 및 감염관리, 낙상예방, 응급상황관리 등의 영역은[24,25] 투약오류로 인한 약물 이상반응과 생리적 손상 예방[13], 병원성 미생물 전파 차단을 통한 의료관련감염 감소[11], 낙상으로 인한 골절 등 신체손상 방지[45], 급성 생리적 악화 상황에서의 신속한 대응[46]과 직접적으로 관련된다. 응급실은 중증도가 높고 생리적 상태가 불안정한 환자가 밀집하여 병원 내 부서 중 안전사고 발생률이 가장 높은 곳으로[5,6], 간호사는 지속적인 모니터링과 직접적인 환자안전간호활동을 통해 환자의 생리적 악화를 조기에 인지하고 예방하는 핵심적인 역할을 수행한다[8]. 이에 간호사의 환자안전간호활동 수준과 영향요인을 규명하는 것은 환자의 신체적 손상 예방, 감염관리, 투약안전 등 환자의 생물학적 안전 보장을 위한 실무 기초자료를 제공한다는 점에서 의의가 있다. 또한 본 연구에서 태도, 주관적 규범, 지각된 행위통제, 행위의도가 환자안전간호활동의 유의한 설명 요인임을 확인함으로써 계획된 행위이론이 응급 간호 실무에서도 유용한 설명 모델임을 확인하였다. 연구결과를 근거로 의사소통 영역의 수행 수준이 가장 낮음을 확인하고 표준화된 지침 마련의 필요성을 제시하였으며, 연령대별 특성을 반영한 교육프로그램 및 조직 차원의 환경적 지원 체계 구축 필요성을 제언하였다는 점에서 간호 실무 및 정책적 의의를 포함한다.

본 연구는 앞서 기술한 의의에도 불구하고 몇 가지 제한점을 가진다. 본 연구는 대전·충청권 소재 7개 종합병원 응급실 간호사를 대상으로 한 횡단적 조사연구로, 연구결과를 전국의 응급실 간호사로 일반화하는 데 한계가 있다. 또한 자기보고식 설문을 사용하여 응답 편향이 개입되었을 가능성이 있으며, 계획된 행위이론 변수 외에 조직문화, 업무 부담 등 응급실 환경의 특수성을 충분히 반영하지 못하였다.

또한 환자안전간호활동과 행위의도는 모두 자기보고식으로 측정함에 따라 두 변수 간 측정상 중첩 가능성을 배제하기 어렵다. 이에 환자안전간호활동은 관찰법이나 체크리스트 활용을 통한 행위 평가 등의 객관적 평가가 병행되어야 할 필요가 있다. 또한 병원 근무 경력과 응급실 근무 경력은 상관관계로 인해 다른 통제변수에 비해 다중공선성 지표(VIF)가 상대적으로 높게 나타났다. 두 변수는 전체 임상경력과 응급실 근무경력이라는 개념적으로 구분되는 정보로 판단하여 모형에 함께 투입하였으나, 상관관계로 인해 개별 계수의 해석에 주의가 필요하다.

한편, 본 연구는 계획된 행위이론의 각 구성요인이 환자안전간호활동에 미치는 개별적 영향력의 크기와 상대적 중요도를 파악하여 실무적 중재의 우선순위를 제시하는 데 목적을 두고 있어 다중회귀분석을 통해 변수의 영향력을 분석하였다. 그러나 다중회귀분석으로 분석하는 경우, 변수 간 위계적 매개 구조를 가정하는 구조방정식모형 또는 경로분석과 달리, 행위의도가 다른 변수들의 설명 변량을 흡수하여 개별 변수의 고유 설명력이 과소 추정될 수 있다는 한계가 있다[40]. 이에 변수 간 선행·후행 관계와 매개 경로를 명확히 규명하기 위해서는 구조방정식모형 또는 경로분석을 적용한 후속 연구가 필요하다.

## 결론

본 연구는 계획된 행위이론을 기반으로 응급실 간호사의 환자안전간호활동에 영향을 미치는 요인을 규명하고자 수행되었다. 연구 결과, 계획된 행위이론은 응급실 간호사의 환자안전간호활동을 설명하는 데 유용한 틀로 확인되었으며, 태도, 주관적 규범, 지각된 행위통제, 행위의도가 주요 영향요인으로 나타났다. 한편 연령은 30세 이상 35세 미만 집단에서만 유의하였고 근무 경력은 유의하지 않았으므로, 연령 및 경력에 따른 차이는 향후 재검증할 필요가 있다. 본 연구결과가 다양한 임상 분야에서 환자안전간호활동 증진을 위한 전략 및 정책 수립과 프로그램 개발에 활용되길 기대한다.

이를 바탕으로 다음과 같이 제언한다. 첫째, 다양한 지역과 규모의 의료기관을 포함한 반복 연구의 수행이 필요하다. 둘째, 자기보고식 설문 외에 관찰 연구나 혼합연구 방법을 활용하여 환자안전간호활동을 보다 객관적으로 평가하는 후속 연구를 제언한다. 셋째, 조직 차원의 환자안전문화, 관리자 지지, 업무 부담 등을 포함한 확장 모형을 적용한 연구가 필요하다.

## Figures and Tables

**Table 1.** Differences in Patient Safety Nursing Activities by General and Job-related Characteristics (N = 152)

Variables	Categories	n (%)	M ± SD	t/F	*p*
Sex	Women	121 (79.6)	4.41 ± 0.44	-1.12	.266
	Men	31 (20.4)	4.51 ± 0.45		
Age (years)	< 30^a^	77 (50.7)	4.28 ± 0.40	10.44	< .001 a < b,c^‡^
	30–34^b^	59 (38.8)	4.55 ± 0.45		
	≥ 35^c^	16 (10.5)	4.68 ± 0.34		
Education level	College	12 (7.9)	4.44 ± 0.50	0.17	.843
	University	127 (83.6)	4.42 ± 0.44		
	≥ Graduate school	13 (8.5)	4.49 ± 0.46		
Marital status	Unmarried	116 (76.3)	4.38 ± 0.43	-2.21	.028
	Married	36 (23.7)	4.57 ± 0.45		
Type of ED	Regional	98 (64.5)	4.40 ± 0.45	-0.93	.352
	Local	54 (35.5)	4.47 ± 0.42		
Hospital work experience (years)	< 5^a^	69 (45.4)	4.34 ± 0.42	8.86	< .001 a,b < c^†^
	5–10^b^	57 (37.5)	4.38 ± 0.43		
	> 10^c^	26 (17.1)	4.74 ± 0.41		
ED work experience (years)	< 5^a^	83 (54.6)	4.34 ± 0.43	5.66	.004 a,b < c^†^
	5–10^b^	54 (35.5)	4.47 ± 0.42		
	> 10^c^	15 (9.9)	4.73 ± 0.44		
Patient safety training experiences within the past year	No	19 (12.5)	4.20 ± 0.52	-2.39	.018
	Yes	133 (87.5)	4.46 ± 0.42		
Number of patient safety training experiences (times)	0	19 (12.5)	4.20 ± 0.52	3.16	.026^‡^
	1	91 (59.9)	4.49 ± 0.45		
	2	29 (19.1)	4.32 ± 0.30		
	≥ 3	13 (8.5)	4.52 ± 0.38		
Awareness of the incident reporting & registration system	No	132 (86.8)	4.43 ± 0.43	0.35	.730
	Yes	20 (13.2)	4.40 ± 0.54		
In the past year, have you ever reported a patient safety accident that you caused?	No	128 (84.2)	4.46 ± 0.45	2.27	.025
	Yes	24 (15.8)	4.24 ± 0.37		
In the past year, have you ever reported a patient safety accident caused by someone else	No	129 (84.9)	4.43 ± 0.45	0.25	.806
	Yes	23 (15.1)	4.41 ± 0.41		

M = Mean; SD = Standard deviation; ED = Emergency department.

† Scheffé test; ^‡^The ANOVA result was significant, but the Scheffé post-hoc test identified no significant pairwise differences among the groups.

**Table 2.** Levels of Attitudes, Subjective Norms, Perceived Behavioral Control, Behavioral Intention, and Patient Safety Nursing Activities (N = 152)

Variables		Range	Item	M ± SD
Attitudes toward PSNA		1–7	4	6.39 ± 0.70
Subjective norms toward PSNA			5	6.48 ± 0.60
Perceived behavioral control toward PSNA			5	5.10 ± 1.10
Behavioral intention toward PSNA			5	5.65 ± 0.98
PSNA	Accuracy of patient identification	1–5	4	4.56 ± 0.54
	Communication		4	4.28 ± 0.65
	Patient safety activities before operations/procedures		3	4.30 ± 0.75
	Fall prevention		6	4.30 ± 0.59
	Hand hygiene and infection prevention		5	4.55 ± 0.49
	Fire safety and emergency management		2	4.37 ± 0.60
	Medication		6	4.57 ± 0.44
	Medical equipment and facilities management		2	4.36 ± 0.73
	Total		32	4.43 ± 0.44

M = Mean; SD = Standard deviation; PSNA = Patient Safety Nursing Activities.

**Table 3.** Correlations among Attitudes, Subjective Norms, Perceived Behavioral Control, Behavioral Intention, and Patient Safety Nursing Activities (N = 152)

Variables	Attitude	Subjective Norms	Perceived Behavioral Control	Behavioral Intention
	r (*p*)	r (*p*)	r (*p*)	r (*p*)
Subjective norms	.63 (< .001)	1	-	-
Perceived behavioral control	.43 (< .001)	.41 (< .001)	1	-
Behavioral intention	.40 (< .001)	.40 (< .001)	.71 (< .001)	1
PSNA	.55 (< .001)	.55 (< .001)	.59 (< .001)	.65 (< .001)

PSNA = Patient Safety Nursing Activities.

**Table 4.** Factors Influencing Patient Safety Nursing Activities (N = 152)

Variables	B	SE	β	t	*p*	Tolerance	VIF
(Constant)	1.24	0.31		3.95	< .001		
Age (30 to < 35 years) (Ref = < 30 years)^†^	0.16	0.06	.18	2.62	.010	.55	1.83
Age (≥ 35 years) (Ref = < 30 years)^†^	0.22	0.12	.15	1.86	.066	.37	2.69
Marital status (Ref = Unmarried)^†^	0.05	0.07	.05	0.75	.453	.55	1.80
Hospital work experience (years)	-0.01	0.01	-.09	-0.95	.346	.26	3.85
Emergency department work experience (years)	0.01	0.01	.11	1.40	.165	.39	2.53
Patient safety training experience (Ref = No)^†^	0.08	0.07	.06	1.15	.250	.84	1.19
Number of patient safety training experiences (times)	0.03	0.02	.10	1.75	.082	.79	1.26
In the past year, have you ever reported a patient safety accident that you caused? (Ref = No)^†^	-0.10	0.06	-.08	-1.57	.120	.93	1.07
Attitudes	0.10	0.04	.16	2.42	.017	.54	1.85
Subjective norms	0.16	0.05	.21	3.20	.002	.55	1.83
Perceived behavioral control	0.06	0.03	.15	1.99	.048	.42	2.36
Behavioral intention	0.17	0.03	.37	5.13	< .001	.47	2.15
R^2^ = .66, Adj R^2^ = .63, Durbin-Watson = 1.88, F = 22.21, *p *< .001							

SE = Standard error; VIF = Variance inflation factor; Ref = Reference.

† Dummy variable.
